# Temperature Regulates Transcription in the Zebrafish Circadian Clock

**DOI:** 10.1371/journal.pbio.0030351

**Published:** 2005-09-27

**Authors:** Kajori Lahiri, Daniela Vallone, Srinivas Babu Gondi, Cristina Santoriello, Thomas Dickmeis, Nicholas S Foulkes

**Affiliations:** **1**Max-Planck Institut für Entwicklungsbiologie, Tübingen, Germany; Wellcome Trust Sanger InstituteUnited Kingdom

## Abstract

It has been well-documented that temperature influences key aspects of the circadian clock. Temperature cycles entrain the clock, while the period length of the circadian cycle is adjusted so that it remains relatively constant over a wide range of temperatures (temperature compensation). In vertebrates, the molecular basis of these properties is poorly understood. Here, using the zebrafish as an ectothermic model, we demonstrate first that in the absence of light, exposure of embryos and primary cell lines to temperature cycles entrains circadian rhythms of clock gene expression*.* Temperature steps drive changes in the basal expression of certain clock genes in a gene-specific manner, a mechanism potentially contributing to entrainment. In the case of the *per4* gene, while E-box promoter elements mediate circadian clock regulation, they do not direct the temperature-driven changes in transcription. Second, by studying E-box-regulated transcription as a reporter of the core clock mechanism, we reveal that the zebrafish clock is temperature-compensated. In addition, temperature strongly influences the amplitude of circadian transcriptional rhythms during and following entrainment by light–dark cycles, a property that could confer temperature compensation. Finally, we show temperature-dependent changes in the expression levels, phosphorylation, and function of the clock protein, CLK. This suggests a mechanism that could account for changes in the amplitude of the E-box-directed rhythm. Together, our results imply that several key transcriptional regulatory elements at the core of the zebrafish clock respond to temperature.

## Abstract 2

Reveals the molecular basis by which temperature cycles entrain circadian rhythms of clock gene expression in zebrafish

## Introduction

The circadian clock plays a central role in adapting the physiology of plants and animals to anticipate day–night environmental changes. Amongst the most conserved properties of the clock is the ability of daily temperature cycles and acute temperature changes to set its phase [1]. In addition, the period length of the clock rhythm remains relatively constant over a wide range of temperatures [1,2]. The mechanism underlying this “temperature compensation” corrects for the natural tendency of the rate of biochemical reactions to change with temperature. Outside of the range of temperature compensation, the clock stops running and arrests at a certain phase [1,3,4]. The physiological range for rhythmicity typically lies well within the temperature range permissive for growth.

In ectotherms, where core body temperature is strongly influenced by the environment, these properties have clear importance to provide a mechanism for daily entrainment of the pacemaker, as well as to ensure that seasonal variations in temperature do not lead to deleterious changes in the speed of the clock cycle [1,5,6]. Although there is homeostatic control of core body temperature in endotherms, recent tissue and cell culture studies have confirmed that their clocks are also temperature-compensated and can be phase-shifted by acute temperature changes [7–9]. In addition, daily rhythms of body temperature have been directly implicated in the maintenance of peripheral clock function [10,11]. Thus, regulation by temperature appears to be a highly conserved property of the circadian timing system.

Molecular studies in a wide range of model organisms have revealed that many clock genes are components of transcription translation feedback loops [12]. For example, in vertebrates, the basic helix-loop-helix Per-Arnt-Sim domain transcription factors, Clock (CLK) and Brain and muscle Arnt-like protein (BMAL), bind as heterodimers to E-box enhancers and activate the expression of other clock genes that encode transcriptional repressors, the Period (Per) and Cryptochrome (Cry) proteins. These repressors interact with CLK-BMAL and interfere with transcriptional activation, thereby reducing expression of their own genes and so closing the feedback loop [13]. Our limited understanding of the molecular basis of temperature responses of the clock has come from studies of the expression of the *period* and *timeless* genes in *Drosophila,* and the *frequency* gene in *Neurospora*. Here, temperature-dependent alterations in transcription [14], mRNA processing [15–17], translation [3,14], protein stability [14,18], and protein–protein interactions [19] have been documented. These results would appear to suggest that unlike the situation for light, multiple regulatory components of the circadian clock mechanism perceive and respond to temperature changes.

Remarkably, little is known about the genetic and molecular basis of the response of the vertebrate clock to temperature. Zebrafish represent an attractive model to explore this issue. Adult fish, larvae, and embryos remain viable over a wide range of core body temperatures (10 °C) that can be regulated accurately by simply controlling the temperature of the water [20]. Unlike other ectothermic vertebrates that have been used in the past to study temperature responses of the clock, zebrafish offer a powerful combination of molecular, genetic, and cell culture tools. Furthermore, the direct entrainment of peripheral clocks in zebrafish by light provides a model to investigate how two zeitgebers integrate to regulate clock function at the cellular level [21,22]. In a recent study of luciferase reporter transgenic zebrafish, Kaneko and colleagues reported that the ambient temperature affects the levels and amplitude of cycling *per3* expression, indicating that temperature can influence clock gene expression in this species [23].

Here we have investigated the effects of temperature on the zebrafish circadian clock in more detail. Specifically, we demonstrate that temperature cycles of as little as 2 °C are sufficient to entrain the zebrafish clock. Temperature steps drive significant changes in the expression levels of several clock genes in a gene-specific manner. In the case of the *per4* gene, while E-box enhancer elements direct circadian expression rhythms, they do not mediate the decreases and increases in expression that immediately follow acute temperature changes. Also, we have studied how temperature affects light–dark (LD) cycle-entrained rhythmic transcription that is directed by the core clock mechanism via E-box elements. We confirm that the zebrafish circadian clock is temperature-compensated. Furthermore, temperature influences the amplitude of rhythmic transcription, a property that could contribute to temperature compensation. The CLK protein expression levels, phosphorylation, and binding to E-box elements, as well as CLK transactivation efficiency, are all temperature-regulated. These temperature-regulated changes in CLK protein function may contribute to the observed changes in rhythm amplitude.

## Results

### Temperature Cycles Influence Clock Gene Expression in Zebrafish Embryos and Cells

**Figure 1:**
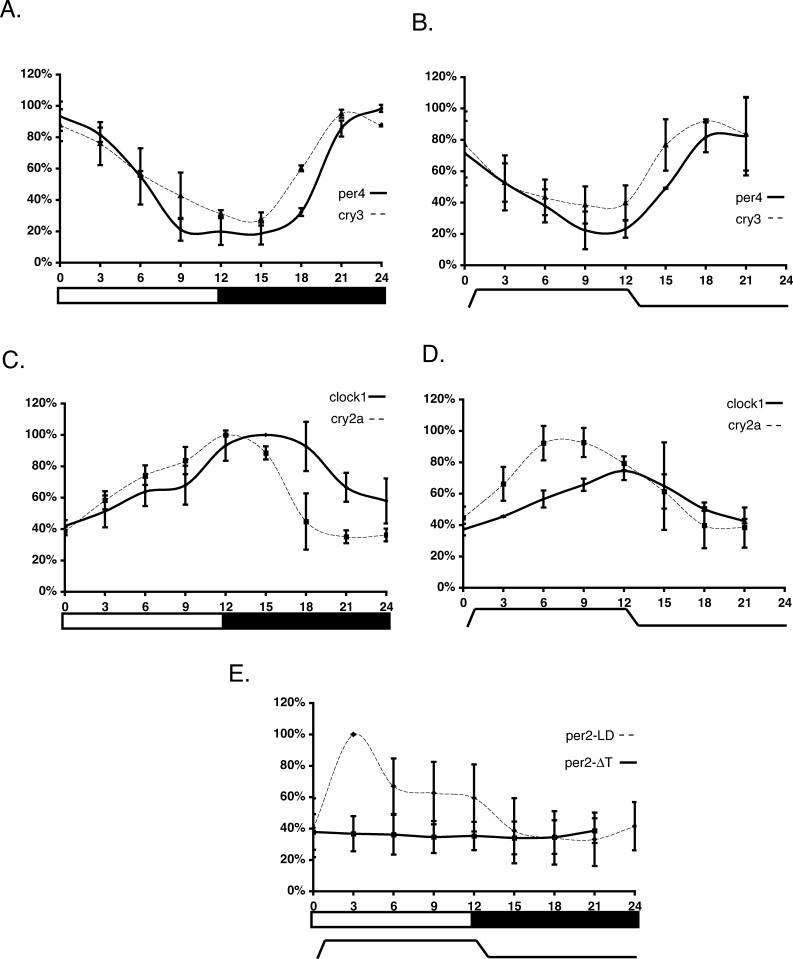
Rhythmic Clock Gene Expression under LD and Temperature Cycles Graphical summary of RPA assays are described: (A) *Per4* (solid line) and *cry3* mRNA expression (dashed line) in zebrafish larvae raised for 6 d either in a light (12 h) or dark (12 h) cycle at a constant temperature (25.3 °C). (B) *Per4* (solid line) and *cry3* mRNA expression (dashed line) in zebrafish larvae raised for 6 d in DD, under a temperature cycle of 4 °C (23.5 °C/11 h, 27.5 °C/11 h, plus 1 h for each heating and cooling phase). RNA samples were harvested during the seventh day (ZT0 is defined as the beginning of the heating and light periods). (C and D) Equivalent analysis of *clock1* (solid line) and *cry2a* (dashed line) expression in (C) LD, and (D) temperature cycle larvae. (E) *Per2* expression was assayed in LD (dashed line) or temperature cycle (ΔT) larvae (solid line). By linear regression analysis, the slope of the ΔT trace has no significant deviation from zero (R^2^ = 0.033 and *p* = 0.66, F-test). The LD cycle curve fits to a 6th-order polynomial regression model (R^2^ = 0.96 and Runs test for deviation from model *p* = 0.99). In each case, zeitgeber time is plotted on the *x*-axis while the relative expression levels (percentage) are plotted on the *y*-axis. *β-actin* levels were used to standardize the results. The highest band intensity in each experiment was arbitrarily defined as 100%, and then all other values were expressed as a percentage of this value. All experiments were performed in triplicate, and error bars denote the standard deviation.

It has been well-documented that exposure to LD cycles is essential for the establishment of circadian rhythms in zebrafish larvae [24,25]. We asked whether, in the absence of light, temperature cycles could also entrain the zebrafish circadian clock. Zebrafish larvae were raised from the late blastula stage (4 h post-fertilization) in constant darkness (DD) for 6 d, while being exposed to a 24-h, 4 °C temperature cycle (between 23.5 °C and 27.5 °C) and were then sacrificed at three hourly time points. Sibling larvae were raised at a constant temperature (25.3 °C) under LD cycle conditions and sacrificed in parallel. We then assayed the daily RNA expression profile of a representative group of core clock gene homologs *(per2* & *4*, *cry2a* & *3,* and *clock1)* [26–29] by RNAse protection assay (RPA). In the case of *per4, cry3, clock1,* and *cry2a,* rhythmic expression was detected under both temperature and LD cycle conditions (Figure 1A–1D). Expression of *per4* and *cry3* peaked at, and just before, the dark–light transition, respectively (Figure 1A) and in the middle of the cold period (Figure 1B). Expression of *clock1* and *cry2a* peaked in the first half of the dark period and at the light–dark transition, respectively (Figure 1C) and at the high–low temperature transition and the middle of the warm period, respectively (Figure 1D). Thus the phase relationship between the expression rhythms of all four genes is preserved under LD and temperature cycles, with the high temperature phase roughly corresponding to the light period. Remarkably, larvae raised in a 2 °C daily temperature cycle gave comparable results (Figure S1A). Thus, exposure of zebrafish to even shallow temperature cycles during early development is sufficient to establish rhythmic clock gene expression. In contrast, while *per2* rhythmic expression is encountered in the LD cycle larvae, low, non-oscillating levels are detected in the temperature cycle conditions (Figure 1E), in agreement with previous reports showing that *per2* expression is light-driven [30,31]. This reveals a differential response of clock gene expression during exposure to LD and temperature cycles.

We have previously demonstrated that zebrafish primary cell lines are powerful in vitro tools to explore the molecular basis of how light entrains the clock in zebrafish peripheral tissues [22,27]. Could these cells also be useful for studying the effects of temperature on the clock? We tested whether exposure to a 4 °C temperature cycle in DD induces rhythmic clock gene expression in the zebrafish PAC-2 cell line. Consistent with our larvae results, we observed an equivalent pattern of clock gene expression in the PAC-2 cells (unpublished data and Figure S1B). Control cells that remained in constant temperature conditions during the entire experiment failed to show expression rhythms. These results indicate that the clock mechanism in zebrafish primary cell lines is able to directly perceive and respond to temperature as well as lighting changes.

### Temperature Cycles Entrain the Zebrafish Circadian Clock

**Figure 2:**
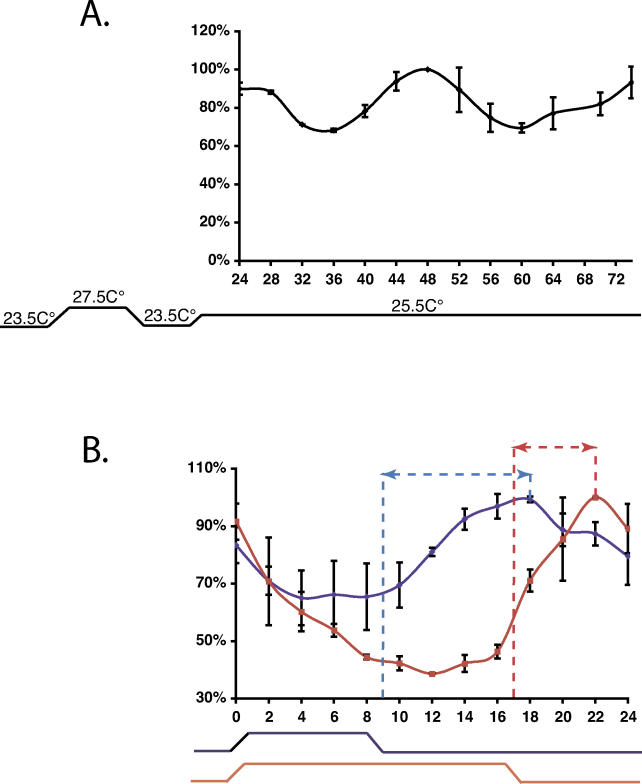
Temperature Cycles Entrain the Zebrafish Circadian Clock (A) Quantification of *per4* expression levels as measured by RPA, in WT PAC-2 cells initially exposed for 6 d to a 4 °C temperature cycle and then transferred to a constant temperature (25.5 °C) for 72 h. RNA extracts were prepared from 24 h to 72 h following transfer to the constant temperature at four hourly intervals. RPA band intensities were quantified and adjusted as described in Figure 1. (B) RPA results quantifying *per4* expression levels in WT PAC-2 cells exposed for 5 d to warm:cold temperature cycles (as indicated below the *x*-axis), including either a 8 h:16 h (blue trace), or a 16 h:8 h (red trace) cycle. The blue and red dashed lines and arrowheads indicate the delay between the warm–cold transition and the peak of *per4* rhythmic expression in each temperature cycle. All experiments were performed in triplicate, and error bars denote the standard deviation.

An important issue is whether temperature cycles drive changes in clock gene expression or if they actually entrain the zebrafish circadian oscillator. We addressed this question in the following ways by examining expression of the *per4* gene in PAC-2 cells. One prediction for entrainment of the oscillator is that circadian rhythms of clock gene expression established by temperature cycles should persist for several cycles following transfer to a constant temperature. We confirmed that a circadian rhythm of endogenous *per4* expression could be measured between 24 h and 72 h after transfer of cells from a 4 °C temperature cycle to constant temperature (Figure 2A). Control cells held at a constant temperature during the entire experiment failed to show cycling gene expression (unpublished data and Figure S1B). We also tested how the phase of clock gene expression is affected by changing the thermoperiod of the temperature cycle (i.e., the relative length of the warm and cold phases of the 24-h cycle). The prediction for entrainment is that the phase of the clock gene rhythm will change relative to the phase of the temperature cycle as a function of the thermoperiod, i.e., a phase angle change [32]. In a temperature-driven response, the phase relationship would be predicted to remain constant; as the cells are warmed or cooled, a change in gene expression should occur immediately, regardless of the thermoperiod. [32]. We exposed PAC-2 cells to 24-h temperature cycles, with either short (8 h) or long (16 h) warm periods and then assayed *per4* expression. The daily expression peak shifts from 9 h after the warm–cold transition in 8:16 (8 h warm:16 h cold) cycles to 5 h after the same transition in 16:8 cycles, and in this way remains consistently locked to the middle of the cold period (Figure 2B). Thus, also this result points to the clock being entrained by temperature cycles. Interestingly, the start of the increase in *per4* expression consistently coincides with the warm–cold transition, with differences in the waveform leading to changes in the timing of the subsequent peak. This suggests that temperature steps can also drive changes in clock gene expression.

### Temperature Shifts Directly Drive Clock Gene Transcription

**Figure 3:**
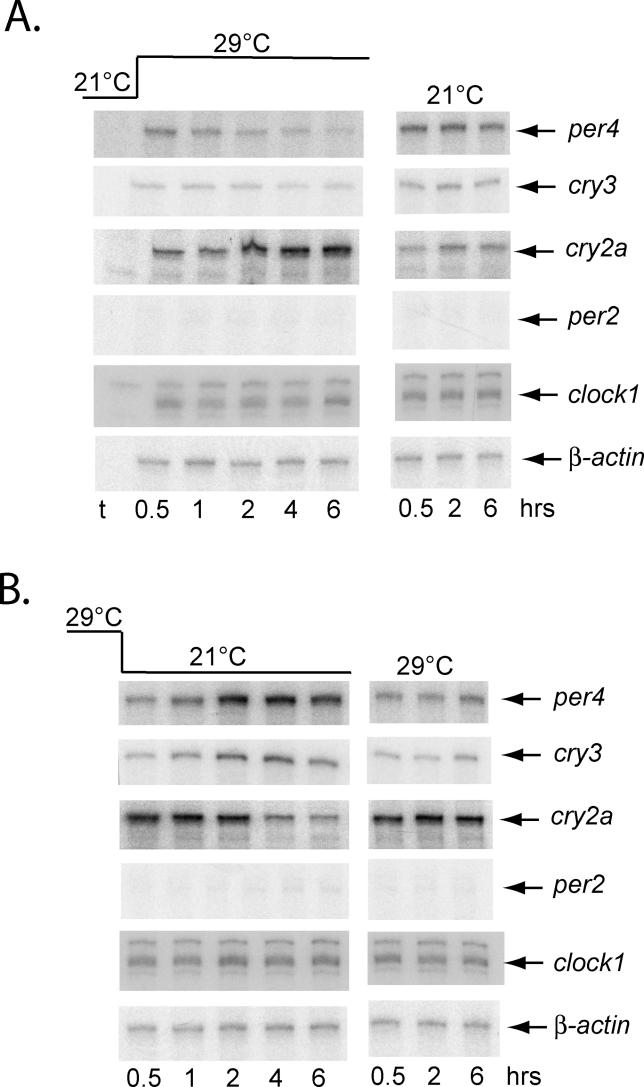
Temperature Steps Regulate Clock Gene Expression Levels (A) Larvae were raised in DD at 21 °C for 7 d and then shifted to 29 °C and harvested at the indicated times relative to the temperature shift (h). Controls remained at 21 °C and were harvested in parallel with the temperature shift larvae. RPA analysis of the indicated genes was then performed. “t” represents a tRNA control sample. (B) As in (A), except that 5-d-old larvae were shifted from 29 °C to 21 °C, and controls remained at 29 °C. All data are representative of at least three independent experiments.

Previous results have demonstrated that temperature steps can elicit acute changes in the expression levels of clock genes in non-vertebrates [14]. Do zebrafish also show this property? We raised larvae at a constant temperature in DD, then abruptly shifted the temperature up or down by 8 °C (21 °C → 29 °C, or 29 °C → 21 °C, respectively), and the resulting expression of a range of clock genes was assayed during the following 6 h (Figure 3A and 3B). Given the strong influence of temperature on the rate of early zebrafish development [20], the larvae at 21 °C were raised for 2 d more than those at 29 °C prior to the temperature step in order to ensure that both sets were at a comparable developmental stage. Expression of *per4* and *cry3* was strongly down-regulated following the temperature increase (3.3 ± 0.4-fold and 1.7 ± 0.2-fold, respectively, at 4 h following the temperature step up), and up-regulated following the temperature decrease (4.7 ± 0.8-fold and 3.6 ± 0.6-fold, respectively, at 4 h following the temperature step down), while the opposite response was observed for *cry2a* (1.8 ± 0.2-fold increase and 2.0 ± 0.3-fold decrease at 4 h following the temperature steps). The temperature steps did not affect the expression of *clock1, per2,* or *β-actin*. Furthermore, no significant changes in expression for any of the genes were detected in control samples that remained at a constant temperature (21 °C or 29 °C) in DD (Figure 3A and 3B). These results reveal that changes in zebrafish clock genes expression do occur following temperature steps, and that this constitutes a gene-specific response.

**Figure 4:**
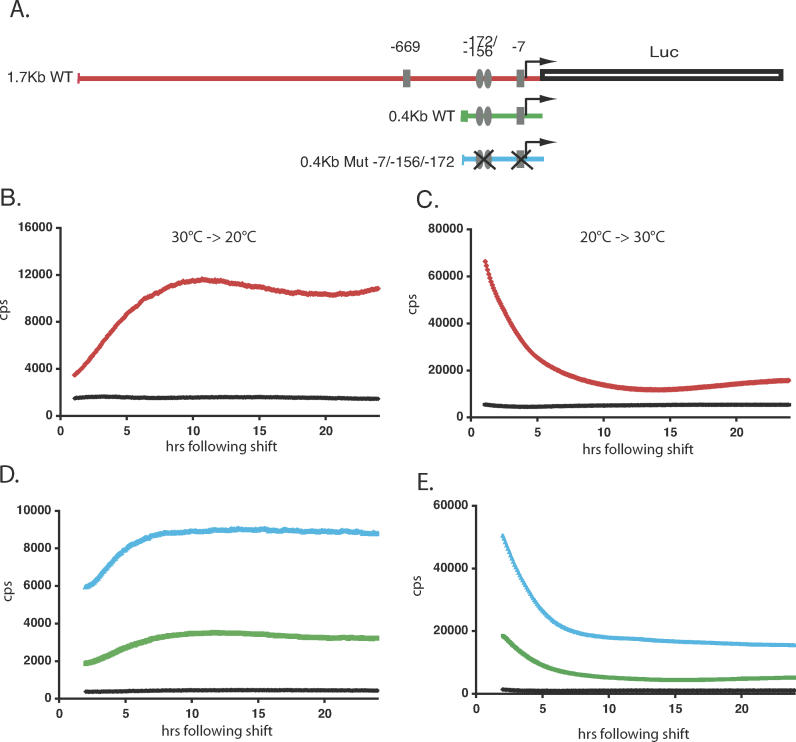
Temperature Steps Induce Changes in *per4* Gene Transcription (A) Schematic representation of the 1.7-kb WT (red), 0.4-kb WT (green), and the 0.4-kb Mut −7/−156/−173 (blue) *per4* promoter luciferase reporter constructs. E-box elements are represented by rectangles ( CACGTG) and ellipses ( AACGTG), and their positions relative to the principal transcription start site are labeled. E-boxes that have been ablated by mutation to the sequence ( CTCGAG) are shown by a cross [27]. (B) Bioluminescence from PAC-2 cells stably transfected with the 1.7-kb WT construct adapted to 30 °C and then shifted rapidly to 20 °C (red trace). During the entire assay, the plate was held inside the Topcount counting chamber, and each well was counted for 3 s at intervals of approximately 5.5 min. Bioluminescence (counts per second) is plotted against time (h) following the temperature shift. A black trace represents pGL3Control transfected cells bioluminescence. (C) Equivalent experiment to that in panel B, with cells adapted to 20 °C and shifted to 30 °C. (D and E) Cells transfected with the 0.4-kb WT and 0.4-kb Mut −7/−156/−173 constructs were subjected to the same rapid temperature decrease and increase, respectively, as described for (B) and (C). All traces represent the mean values of 16 independent wells. Each panel is representative of at least three independent experiments.

To explore in more detail the mechanism underlying the changes in gene expression that follow temperature steps, we focused our attention on *per4,* a gene where we have previously studied the regulation by the circadian clock and light [27]. Do temperature steps affect the levels of *per4* transcription? We investigated the effect of temperature steps on the expression of a 1.7-kilobase (kb) *per4* promoter–luciferase reporter construct in stably transfected PAC-2 cells (1.7-kb wild-type [WT], Figure 4A) [27]. As a control for the direct effect of temperature on the rate of the luciferase-catalyzed bioluminescent reaction, in parallel we analyzed cells stably transfected with a luciferase reporter construct driven by the SV40 promoter and enhancer sequences (pGL3 Control). We previously confirmed that expression of this viral promoter reporter construct is neither regulated by the circadian clock nor by changes in temperature (unpublished data). Cells were exposed for 5 d in DD to 30 °C or 20 °C, then the temperature was decreased (30 °C to 20 °C, Figure 4B) or increased (20 °C to 30 °C, Figure 4C), and subsequently bioluminescence was assayed for 24 h. A temperature decrease resulted in sustained induction (3.34 ± 0.63-fold increase) (Figure 4B), while a temperature increase led to significant down-regulation of *per4* promoter expression (5.40 ± 0.21-fold decrease) (Figure 4C) during the first 11 h following the temperature steps, relative to the pGL3 Control. Expression subsequently remained at these new levels for the remainder of the analysis (Figure 4B and 4C). Using RPA analysis, we confirmed that increases and decreases of luciferase mRNA expression occur following the temperature steps in the 1.7-kb WT transfected cells (Figure S2). These results reveal that a transcriptional mechanism is at least in part responsible for the changes in expression that follow temperature steps.

Do these temperature-dependent changes in transcription use the same pathway or regulatory elements that mediate the clock regulation of *per4,* i.e., the E-box enhancer elements? We have previously demonstrated that mutation of E-box elements within the *per4* promoter eliminates circadian clock control [27]. Therefore, we compared the acute temperature response of a 0.4-kb *per4* promoter luciferase reporter construct containing three E-box elements (0.4-kb WT, Figure 4A) with the same construct where the sequences of all E-box elements have been mutated [0.4-kb mutant (Mut) −7/−156/−173]. The mutation of the E-box elements did not eliminate the increases (1.5 ± 0.16-fold) or decreases (3.0 ± 0.11-fold) in expression following temperature steps that were observed in the 0.4-kb WT construct (1.9 ± 0.17-fold increase and 4.1 ± 0.12-fold decrease) (Figure 4D and 4E). Thus, the results reported here demonstrate that promoter elements distinct from E-boxes, within 0.4 kb of the *per4* transcription start site, are responsible for driving the changes in expression in response to temperature steps.

### The Zebrafish Circadian Clock Is Temperature-Compensated

**Figure 5:**
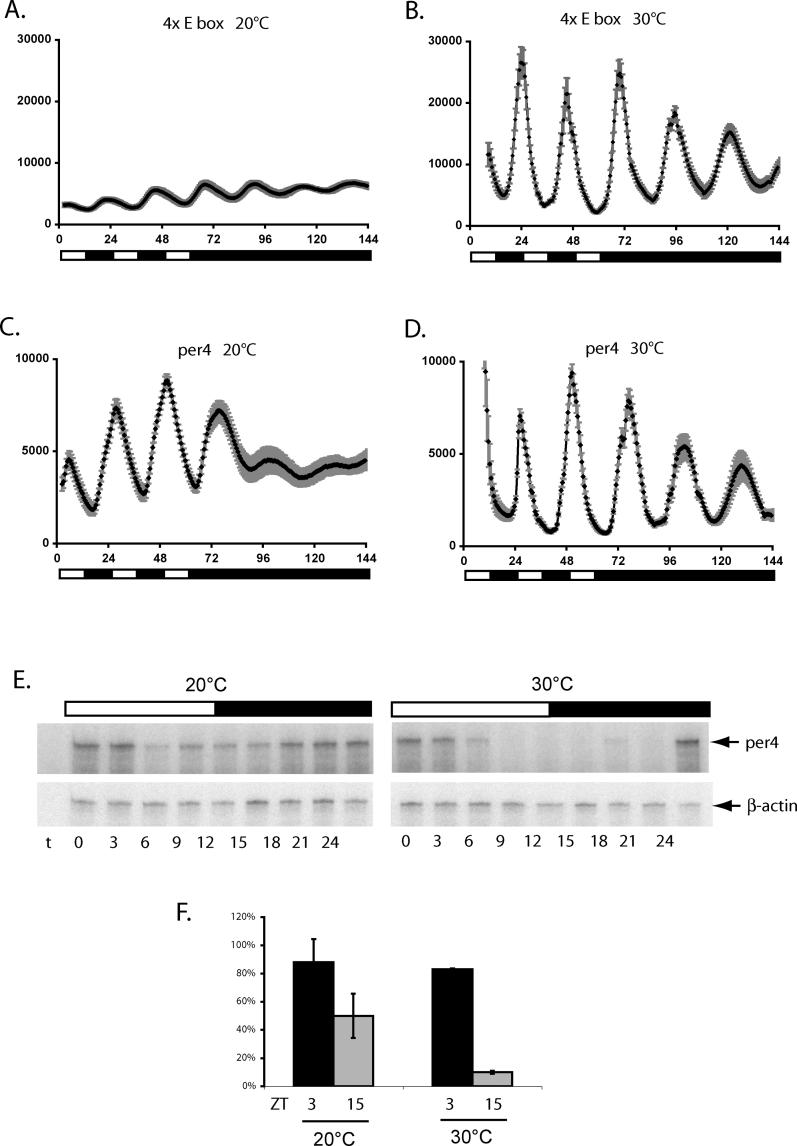
Temperature Compensation and the Amplitude of E-box-Directed Rhythmic Expression (A) Bioluminescence profile of 4xE-box (−7) reporter cells held at 20 °C under a LD cycle and then transferred to DD conditions. Plates were counted once per hour and maintained in robotic stacking units between assays, where they were illuminated. (B) Equivalent experiment to panel A, with cells maintained at 30 °C. (C) Bioluminescence traces from 1.7-kb WT *per4* reporter cells maintained at 20 °C under LD cycle and DD conditions. (D) Bioluminescence traces from 1.7-kb WT *per4* reporter cells maintained at 30 °C under LD cycle and DD conditions. (E) RPA analysis of *per4* expression in WT PAC-2 cells held at 20 °C and 30 °C under an LD cycle for 3 d. RNA extracts were prepared on the fourth day at 3-h intervals during one 24-h cycle. Time 0 represents ZT 0: the onset of the light period. A white and black bar above the autoradiograph indicates the duration of the light and dark periods. RPA results with a β-actin loading control are also shown. “t” represents a tRNA control sample. (F) A bar graph shows quantification of the peak (ZT3) and trough (ZT15) *per4* expression values at 20 °C and 30 °C plotted as described in Figure 1, with error bars representing the standard deviation of three independent experiments. All bioluminescence traces represent the mean values of 16 independent wells. Each panel is representative of at least three independent experiments.

An essential property of the zebrafish circadian clock is that it accurately measures time over a range of temperatures. Temperature-dependent changes in core clock function may be predicted to compensate for the tendency of the speed of the oscillator to vary with the ambient temperature [1,2]. We initially wished to confirm that the zebrafish clock is temperature-compensated. We measured the period length of circadian-clock-generated rhythms entrained by LD cycles as a function of temperature, in two luciferase reporter constructs. The first construct contained four E-box elements within their natural context of the *per4* promoter (1.7-kb WT), while the second was a heterologous promoter containing four copies of a *per4* E-box element cloned upstream of a synthetic TATA box [4xE-box (−7)]. The reporter cells were assayed during incubation at a constant 20 °C, 25 °C, or 30 °C (Figure 5 and [27]) for 3 d in LD cycles and then transferred to DD for 3 d. We measured the period length of the rhythmic luciferase expression between the second and third day in DD. The free-running period length (τ) for the E-box heterologous promoter was 22.98 ± 0.17 h at 20 °C, 25.19 ± 0.21 h at 25 °C, and 25.46 ± 0.15 h at 30 °C, predicting that the temperature coefficient Q_10_ = 0.9 over the range 20 °C to 30 °C (see Materials and Methods, and Figure 5A and 5B). This confirms that the zebrafish clock indeed shows temperature compensation over a 10 °C range. However, calculations of period length were more problematic for the *per4* promoter reporter, particularly at 20 °C, due to its very rapid dampening in DD conditions (Figure 5C and 5D). Interestingly, the bioluminescence traces of both constructs showed a pronounced change in rhythm amplitude between 20 °C (Figure 5A and 5C) and 30 °C (Figure 5B and 5D), both in LD and free running conditions [for 1.7-kb WT, 12.1 ± 1.6-fold at 30 °C, and 3.3 ± 0.56-fold at 20 °C, and for 4xE-box (−7) 9.7 ± 1.0-fold at 30 °C, and 1.6 ± 0.11-fold at 20 °C as measured on the second d in LD]. To confirm that this also occurs in the case of the endogenous *per4* gene, we examined *per4* RNA expression in cells entrained by an LD cycle at a constant 20 °C or 30 °C (Figure 5E and 5F). A higher-amplitude rhythm of expression was observed at 30 °C (7.9 ± 0.56-fold difference between peak and trough), compared with that at 20 °C (1.91 ± 0.5-fold). Thus, at least in part, the ambient temperature strongly influences the amplitude of circadian rhythms of transcription, during and following entrainment by LD cycles. Such a property has been already proposed by mathematical models to explain temperature compensation of the circadian clock [33–35].

### E-box Function Is Influenced by Temperature

**Figure 6:**
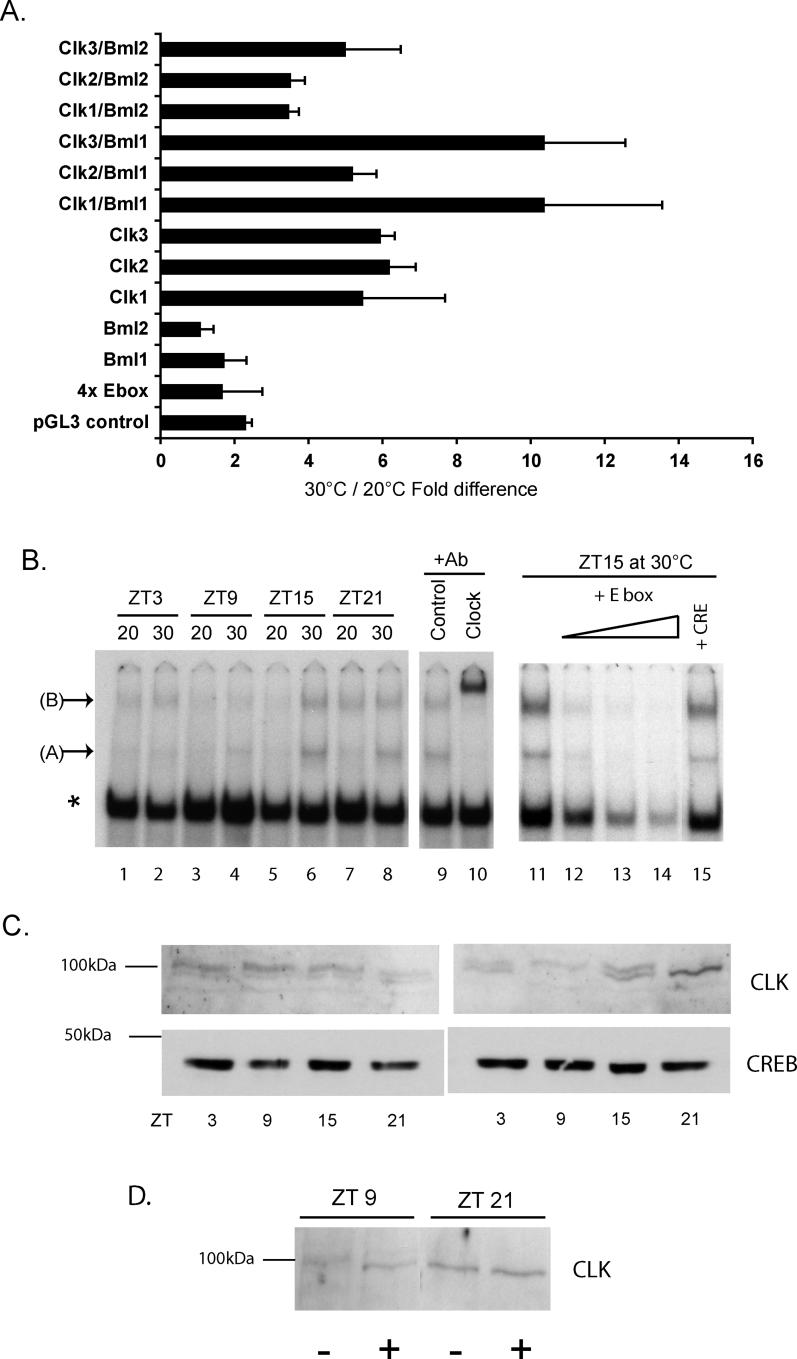
Temperature Influences CLK Protein Expression and Function (A) In vitro luciferase assays of transiently transfected PAC2 cells. The combinations of CLK (Clk) and BMAL (Bml) expression vectors cotransfected with the 4x Ebox (−7) reporter plasmid are indicated for each assay result. Control cells were transfected with the reporter plasmid or with the pGL3 Control plasmid alone. Values represent the mean fold difference between luciferase activities measured in 30 °C and 20 °C, 60 h after transfection. All assays were standardized for transfection efficiency using a β-galactosidase assay. The results are based on four independent experiments, and error bars indicate the standard deviation. (B) Electrophoretic mobility shift assay of nuclear extracts from PAC-2 cells cultured at 20 °C or 30 °C on a LD cycle, and harvested at ZT3, 9, 15, and 21 (lanes 1 to 8). Three specific complexes are indicated by A, B, and an asterisk. Supershift assays of a ZT15, 30 °C extract (+Ab), used either a dopamine transporter antibody (Control) or a mouse clk antibody (Clock) (lanes 9 and 10). Complexes indicated by A, B, and an asterisk are all efficiently competed by a 25-, 50-, and 100-fold excess of cold E-box probe (lanes 12, 13, and 14, respectively, and compare with lane 11), but not with a 100-fold excess of a CRE probe (compare lane 15 with lane 11). (C) Western blotting assay using the anti-mouse CLK antibody of the same nuclear extracts tested in the electrophoretic mobility shift assay analysis of panel B. The migration of a 100-kDa marker band is shown. Below are shown western blotting results for the same extracts using an anti-mouse CREB antibody as a loading control. (D) Western blot assay of CLK protein in 30 °C extracts prepared at ZT9 or ZT21 (time points representing the trough and peak, respectively, of the CLK protein rhythm). Samples were prepared with (+) or without (−) treatment with alkaline phosphatase prior to electrophoresis and transfer. In panels B, C, and D, data are representative of at least three independent experiments.

What makes the amplitude of E-box-driven rhythmic expression respond to temperature? The basic helix-loop-helix Per-Arnt-Sim domain clock proteins, CLK and BMAL, bind to E-boxes as heterodimers and thereby activate transcription at certain phases of the circadian clock cycle [13]. Thus, to test whether transcriptional activation mediated by CLK and BMAL was temperature dependent, we transiently transfected the 4xE-box (−7) luciferase reporter plasmid together with various combinations of zebrafish CLK1, 2, and 3, and BMAL1 and 2 expression constructs. We compared the levels of activation between cells incubated at 20 °C and 30 °C (Figure 6A). At 30 °C, activation driven by all three CLK family members alone was 5- to 6-fold higher than at 20 °C. Co-transfection of CLK1 and CLK3 in combination with BMAL1 led to an even higher activation at 30 °C relative to 20 °C (6- to 10-fold). In contrast, the activation obtained by transfecting BMAL 1 or 2, the reporter construct alone, or the pGL3Control plasmid was only 1.5- to 2-fold higher at 30 °C than at 20 °C. Thus, the amplitude of transcriptional activation driven by the members of the CLK family, alone or in combination with BMAL1, appears to be strongly temperature-dependent in zebrafish cells.

Does temperature influence the binding of endogenous CLK protein-containing complexes to circadian E-boxes? Nuclear extracts were prepared at specific time points from cells maintained under a LD cycle, at 20 °C or 30 °C and then tested for binding to an E-box probe containing two consensus E-box sequences, by electrophoretic mobility shift assay (Figure 6B). Interestingly, levels of two slow mobility complexes varied according to the temperature and the time of day. Levels of one complex (complex A) in 30 °C cells followed a 24-h rhythm with peak levels at Zeitgeber Time (ZT) ZT15 and a trough at ZT3 (Figure 6B, lanes 6 and 2, respectively). In contrast, at 20 °C this complex was barely detectable at all time points. At 30 °C, levels of a second complex (complex B) showed a rhythm similar to that observed for complex A, while at 20 °C peak levels were reduced and shifted to ZT21 (lanes 1, 3, 5, and 7). Levels of a third abundant complex (indicated by an asterisk) did not change significantly according to the time of day or temperature. In control experiments, the binding of all three complexes at ZT15 at 30 °C were efficiently competed by an excess of cold E-box sequence (lanes 11–14) but not by an unrelated cAMP response element sequence (lane 15). Thus, it appears that temperature affects the levels of certain specific E-box-binding complexes. In order to test whether CLK proteins are components of these complexes, we performed a supershift assay using a mouse CLK-specific antibody (Figure 6B). This antibody efficiently supershifted both complexes A and B, but not the abundant complex (indicated by an asterisk) (lane 10), while a control antibody (anti-dopamine transporter) failed to supershift any of the complexes (lane 9). To test which of the three zebrafish CLK proteins were recognized by this mouse antibody, myc-tagged versions of the CLK proteins were expressed in an in vitro translation system and were then analyzed by western blotting. While all zebrafish CLK proteins were efficiently recognized by the control myc tag antibody, only CLK1 (and, to a lesser extent, CLK3) were detected by the mouse CLK-specific antibody (Figure S3).

Do the levels of CLK protein change at the high and low temperatures? We performed a western blot analysis of the same nuclear extracts prepared for the electrophoretic mobility shift assay using the mouse CLK-specific antibody. This assay revealed, at all four (20 °C) time points, the presence of two closely spaced bands, similar in size to that predicted for the CLK1 protein (approximately 100 kDa, Figure 6C). The overall levels of CLK-immunoreactive bands were comparable at the four time points; however, at ZT21 the two bands showed an equal intensity while at ZT9 the high molecular weight band was stronger. In contrast, at 30 °C, overall CLK protein levels changed during the 24-h cycle, with peak levels at ZT21 and a trough at ZT9 (Figure 6C). Also, at 30 °C, the relative levels of the two bands changed considerably between the four time points. Specifically, the intensity of the higher molecular weight band decreased significantly between ZT9 and ZT21, while the intensity of the lower molecular weight band increased (Figure 6C).

Does this result reflect temperature-dependent changes in CLK post-translational modifications? The phosphorylation status of several clock proteins in various model systems has been shown to vary through the circadian cycle, a property linked with changes in the protein stability or function [36–38]. In many cases, different levels of phosphorylation can be visualized by changes in electrophoretic mobility of the protein. We tested whether the multiple CLK-immunoreactive bands represented various phosphorylated forms of this protein. Extracts from the peak (ZT21) and trough (ZT9) points in the 30 °C cell extracts were treated with phosphatase before western analysis in parallel with untreated controls. Phosphatase treatment of the ZT9 extract increased the mobility of the CLK band so that it co-migrated with the single CLK-immunoreactive band in the ZT21 extract (Figure 6D). The mobility of the ZT21 extract band did not alter with phosphatase treatment. This result points to temperature affecting the amplitude of cycling CLK protein levels and their phosphorylation.

## Discussion

The zebrafish, as an ectotherm, represents an ideal vertebrate model system to study the effects of temperature on circadian clock function. Given the geographical distribution, and the shallow, fresh-water habitats of natural populations of zebrafish (data from http://www.fishbase.org, it seems likely that their core body temperature would naturally be subjected to a day–night rhythm, as well as seasonal changes. It is therefore reasonable to predict that temperature would normally play a role in regulating the circadian timing system. Our results have highlighted four responses of the zebrafish clock to temperature: (1) entrainment by even shallow temperature cycles, (2) regulation of the expression levels of many clock genes by temperature steps in a gene-specific manner, (3) temperature compensation, and (4) a strong effect of the ambient temperature on the amplitude of cycling expression of certain clock genes. Furthermore, our observation that the clock in cell lines responds directly to temperature reinforces the notion of autonomy in zebrafish peripheral clock entrainment and provides a valuable cell culture tool to explore the temperature response [22,27].

### Molecular Mechanisms for Entrainment by Temperature

**Figure 7:**
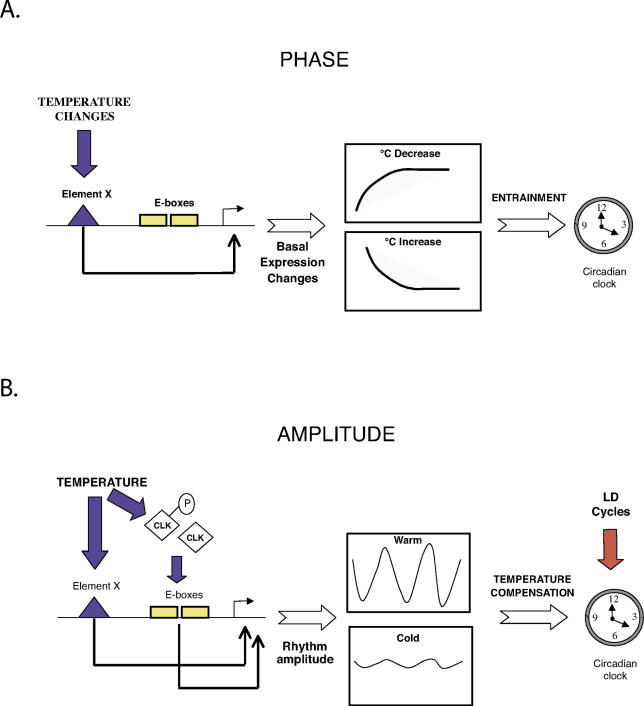
Model for Temperature Regulation of the *per4* Promoter (A) Temperature steps entrain the phase of the clock by driving expression levels of *per4* and other clock genes via a hypothetical enhancer element X. Temperature decreases result in expression increases, and vice versa. Although E-boxes ultimately mediate regulation of the *per4* promoter by the entrained clock, they do not participate in the temperature-driven response. (B) Temperature influences the amplitude of rhythmic *per4* expression that has been entrained by LD cycles in two ways: (1) by determining the amplitude of E-box-directed rhythmic expression, via changes in CLK protein levels, phosphorylation, and E-box binding, and (2) by driving expression changes through element X (see panel A). The promoter integrates these two regulatory mechanisms. The temperature-dependent amplitude of E-box-directed rhythmic expression would be predicted to involve the core feedback loops of the clock itself and, according to mathematical models, might thereby underlie temperature compensation.

Our results clearly point to temperature cycles entraining rhythms of circadian gene expression in zebrafish. We base this conclusion on two observations: (i) *per4* expression rhythms persist after transfer from temperature cycles to constant temperature, and (ii) the phase of the *per4* rhythm changes relative to the phase of the temperature cycle as a function of the length of the thermoperiod. However, the response of the *per4* rhythm to temperature cycles with different thermoperiods suggests that temperature changes could also drive expression of certain clock genes (the increase in *per4* expression consistently coincides with the warm–cold transition). Indeed, we show that acute temperature steps significantly alter the expression levels of several clock genes in a gene-specific manner, not simply reflecting global changes in transcription rate. In the case of the *per4* promoter, these changes do not involve circadian clock regulation via E-boxes, suggesting a temperature-driven response (see scheme in Figure 7A). The acute responses observed for *per4, cry3,* and *cry2a* after the temperature steps match their expression profile, following the individual temperature transitions under temperature cycle conditions. Thus, we speculate that the acute temperature regulation of these genes may contribute to entrainment of the clock mechanism and so represents a component of the temperature input pathway (Figure 7A). Within this input pathway, the gene expression response may lie downstream of more rapid temperature-dependent regulatory events that also contribute to entrainment of the clock. The *per4* gene expression rhythm observed under temperature cycles may represent the integration of the temperature-driven response (via element X) and regulation by the entrained circadian clock (via E-boxes). Further studies will be required to test these hypotheses.

An ectothermic organism needs to modify many aspects of its physiology in order to adapt to substantial changes in core body temperature. The presence of circadian and circannual clocks and the ability to respond to photoperiodic changes provide mechanisms to anticipate regular daily and seasonal temperature changes and so give sufficient time to mount an appropriate gene expression response [39]. However, many studies have shown that changes in gene expression also occur at the cellular level in direct response to temperature alterations [39–41]. The transcriptional mechanism that regulates *per4* expression following temperature shifts may therefore constitute a more general mechanism whereby cells perceive and adapt to temperature changes.

By comparing gene expression under LD and temperature cycle conditions, we have confirmed previous reports that *per2* expression is light driven [30,31] and now show that this clock gene is not induced by all clock-entraining signals (zeitgebers). Thus, light and temperature cycles appear, at least in part, to drive gene expression within the circadian clock by distinct pathways. The presence of larger clock gene families in teleosts may have led to specialization of individual genes to respond to single zeitgebers [25].

### Temperature Regulates the Amplitude of Rhythmic Clock Gene Expression: A Mechanism Underlying Temperature Compensation?

Temperature compensation of the circadian clock is essential to preserve its timing function over a range of temperatures [1]. We have confirmed that the zebrafish PAC-2 cell clock is temperature-compensated, actually decreasing the rate of its oscillation slightly when the temperature is increased, as has been reported for other cell culture model systems [7,8]. Mathematical models in which temperature influences the amplitude of the circadian pacemaker have been proposed to explain various aspects of the behavior of circadian clock outputs including their temperature compensation [33–35]. Consistently, we observe that the amplitude of circadian E-box-directed rhythmic transcription entrained by LD cycles is 6-fold higher at 30 °C than at 20 °C, while the phase remains constant. The temperature also influences the amplitude of *per4* rhythmic expression. However, the changes in the amplitude of the E-box heterologous promoter rhythm originate from differences in the peak values, while for the *per4* promoter, the amplitude is determined by differences in the trough values. To explain this apparent discrepancy, we propose a model (Figure 7B) where the *per4* promoter can integrate temperature and light regulatory input from the E-boxes together with regulation by other temperature-driven elements (X in our model). This ultimately results in the trough levels of expression being set by the E-box-independent, temperature-driven regulation (higher trough levels at lower temperatures). Instead, the *relative* levels of the peaks respond to E-box input (peak values remain constant since the amplitude of light-cycle-entrained rhythmic expression decreases at lower temperatures) (Figure7B).

We have explored the mechanism whereby the amplitude of E-box-directed expression rhythms respond to the temperature. The CLK-directed activation of an E-box reporter construct is 5- to 10-fold higher at 30 °C than at 20 °C as measured in transfection assays. This strong effect of temperature on transcriptional activation appears to be CLK-specific, because not only is E-box reporter expression driven by BMAL alone, expression of control reporter constructs differs by only 1.5- to 2-fold over the same 10 °C range. Our studies using PAC-2 cells have also revealed that the levels of endogenous CLK-containing nuclear complexes that bind specifically to E-boxes as well as CLK protein levels and phosphorylation change as a function of temperature and time of day during entrainment by a LD cycle. We speculate that these properties may ultimately define the E-box rhythm amplitude (Figure 7B). Indeed, regulation of the phosphorylation of clock proteins has been tightly linked with other basic properties of the circadian clock [36–38].

Looking from a broader perspective, the temperature-responsive transcriptional regulatory mechanisms that we have revealed in this study may form part of more general mechanisms that directly adapt gene expression and cell physiology to changes in ambient temperature. Thus the implications of our work may reach beyond the circadian clock.

## Materials and Methods

### RNA and protein analysis

RNA extractions from larvae and cells, RPAs, and the *per4, β-actin,* and *clock1* riboprobes have been described [27,28]. The remaining riboprobes were for *per2* [26,42] (from positions 3113–3874 relative to the translation start site), for *cry2a* [29] (positions 1589–1968), and for *cry3* [29] (positions 1339–1797). The *clock* cDNAs were transcribed and translated using the TnT-Quick Coupled Transcription/Translation System (Promega, Madison, Wisconsin, United States), before western blotting (BioRad, Hercules, California, United States) using an anti-mouse CLK (Santa Cruz Biotechnology, Santa Cruz, California, United States) or myc antibody (Upstate Biotechnology, Lake Placid, New York, United States), and visualization with the ECL detection system (Amersham Biosciences, Little Chalfont, United Kingdom). For phosphatase treatments, nuclear extracts were prepared as described for the electrophoretic mobility shift assays, without the addition of phosphatase inhibitors. Extracts were then treated with 1 unit of calf intestinal phosphatase (Roche, Basel, Switzerland) in nuclear extract buffer, at 37 °C for 15 min. Laemmli buffer was added to a final concentration of 1× and the samples heated at 95 °C for 5 min before SDS electrophoresis and western blotting analysis. The polyclonal anti-mouse CREB antibody was purchased from Cell Signaling Technology (Beverly, Massachusetts, United States).

### Transient transfection assays

A standard electroporation method was used [27]. Transfected cells were assayed using an in vitro luciferase assay system (Promega). The CLK and BMAL expression constructs were based on the pcDNA3.1/Myc-His expression vector (Invitrogen, Carlsbad, California, United States). Co-transfection with the plasmid pcDNA3.1/Myc-His (+)/lacZ and a β-galactosidase assay served to control for transfection efficiency.

### Electrophoretic mobility shift and supershift assays

Preparation of nuclear extracts, radioactive labeling and purification of oligonucleotide probes, and EMS assays were performed as described elsewhere [43]. The E-box probes contained a tandem repeat of the *per4* promoter E-box (−7) sequence (sense oligo: 5′- GAAGCACGTGTACTCGGAAGCACGTGTACTCG-3′) [27]. Supershift assays using an anti-mouse CLK and dopamine transporter antibodies (Santa Cruz Biotechnology) were performed as described elsewhere [43].

### Cell cultures and in vivo luciferase assays

Culture conditions and in vivo luciferase assays have been described [27]. In 0.4-kb Mut −7/−156/−172 contains a *per4* promoter fragment, extending between −207 and +190 relative to the transcription start site, cloned in pGL3Basic where the E-box sequences are mutated to CTCGAG by site-directed mutagenesis.

### Raising zebrafish larvae, temperature, and lighting control

The zebrafish Tübingen strain was maintained and crossed using standard methods [20]. Flasks containing cells or larvae were submerged in 60-l water baths with circulating heating and cooling units (Lauda, Lauda-Königshofen, Germany) and illuminated with a tungsten light source (11 μW/cm^2^) [24]. Temperature cycles were generated by controlling the heating and cooling units using Wintherm plus software (Lauda).

### Data analysis

Bioluminescence data were analyzed using Microsoft Excel or CHRONO software [27,44]. Period estimates measured after 2 d in DD were made by linear regression following peakfinder analysis with CHRONO [44]. For Q_10_ temperature coefficient calculations, period length estimates for cells held at 20 °C, 25 °C, and 30 °C were calculated as cycles per hour and then plotted against temperature. Linear regression analysis revealed a good fit to a straight line (R^2^ = 0.9734). Mean period lengths at 20 °C and 30 °C were then substituted into the equation Q_10_ = (R_2_/R_1_)^10/(T2-T1)^, where R is rate and T is temperature. Autoradiographic images were quantified with the aid of Scion Image software (NIH, http://rsb.info.nih.gov/nih-image/). Statistical analysis was performed with the aid of GraphPad PRISM 4 software (GraphPad Software, San Diego California, United States).

## Supporting Information

Figure S1 Temperature Cycles Induce Rhythmic *per4* mRNA Expression in Larvae and PAC-2 Cells (A) RPA analysis of *per4* and *β-actin* expression in larvae raised for 7 d in DD on a 2 °C temperature cycle (24 °C/11.5 h, 26 °C /11.5 h, plus an additional 0.5 h for both heating and cooling phases). During the seventh day, RNA was harvested at the indicated times (Time 0 is defined as the beginning of the heating period).
(B) RPA analysis of *per4* and *β-actin* expression in PAC-2 cells cultured for 7 d in a 4 °C temperature cycle (23.5 °C/11 h, 27.5 °C/11 h, plus 1 h for each heating and cooling phase) under DD conditions. Cells were harvested during the seventh day. Control cells maintained at a constant temperature (25 °C) during the entire experiment were harvested and assayed in parallel.
(2.16 MB TIF).

Figure S2 Temperature Steps Induce Changes in *Luciferase* Reporter mRNA Expression (A) Quantification of RPA analysis of *luciferase* mRNA expression in 1.7-kb WT stably transfected cells, 0, 1, 3, and 6 h following transfer from 30 °C to 20 °C. The experiment was performed in triplicate, and error bars denote the standard deviation.
(B) Equivalent analysis of *luciferase* expression in cells transferred from 20 °C to 30 °C.
(512 KB TIF).

Figure S3 Analysis of Recombinant Zebrafish CLOCK Proteins Western blotting analysis of in vitro transcription/translation extracts containing myc-tagged CLOCK proteins (Clock-myc 1, 2, and 3). Blots were treated with an anti-myc tag monoclonal antibody (myc-Ab) or an anti-mouse CLK polyclonal antibody (Clock-Ab).
(2.24 MB TIF).

### Accession Numbers

The GenBank (http://www.ncbi.nlm.nih.gov/Genbank) accession numbers for the genes and gene products discussed in this paper are *clock1* (AF133306), *clock2* (AB087255), *clock3* (AB087256), *cry2a* (AB042250), *cry3* (AB042252), and *per2* (AY171100).

## Abbreviations

BMAL: Brain and muscle Arnt-like protein
CLK: clock
Cry: cryptochrome
DD: constant darkness
kb: kilobase
LD: light–dark
Mut: mutant
Per: period
RPA: RNase protection assay
WT: wild-type
ZT: Zeitgeber Time
